# Large Language Model–Based Virtual Patient Systems for History-Taking in Medical Education: Comprehensive Systematic Review

**DOI:** 10.2196/79039

**Published:** 2026-01-02

**Authors:** Dongliang Li, Syaheerah Lebai Lutfi

**Affiliations:** 1 Artificial Intelligence & Software Engineering School of Computer Sciences Universiti Sains Malaysia Penang Malaysia; 2 Medical Informatics Department College of Medicine and Health Sciences Sultan Qaboos University Al Seeb Oman

**Keywords:** large language model, virtual patient, medical education, history-taking, simulated patients

## Abstract

**Background:**

Large language models (LLMs), such as GPT-3.5 and GPT-4 (OpenAI), have been transforming virtual patient systems in medical education by providing scalable and cost-effective alternatives to standardized patients. However, systematic evaluations of their performance, particularly for multimorbidity scenarios involving multiple coexisting diseases, are still limited.

**Objective:**

This systematic review aimed to evaluate LLM-based virtual patient systems for medical history-taking, addressing four research questions: (1) simulated patient types and disease scope, (2) performance-enhancing techniques, (3) experimental designs and evaluation metrics, and (4) dataset characteristics and availability.

**Methods:**

Following PRISMA (Preferred Reporting Items for Systematic Reviews and Meta-Analyses) 2020, 9 databases were searched (January 1, 2020, to August 18, 2025). Nontransformer LLMs and non–history-taking tasks were excluded. Multidimensional quality and bias assessments were conducted.

**Results:**

A total of 39 studies were included, screened by one computer science researcher under supervision. LLM-based virtual patient systems mainly simulated internal medicine and mental health disorders, with many addressing distinct single disease types but few covering multimorbidity or rare conditions. Techniques like role-based prompts, few-shot learning, multiagent frameworks, knowledge graph (KG) integration (top-k accuracy 16.02%), and fine-tuning enhanced dialogue and diagnostic accuracy. Multimodal inputs (eg, speech and imaging) improved immersion and realism. Evaluations, typically involving 10-50 students and 3-10 experts, demonstrated strong performance (top-k accuracy: 0.45-0.98, hallucination rate: 0.31%–5%, System Usability Scale [SUS] ≥80). However, small samples, inconsistent metrics, and limited controls restricted generalizability. Common datasets such as MIMIC-III (Medical Information Mart for Intensive Care-III) exhibited intensive care unit (ICU) bias and lacked diversity, affecting reproducibility and external validity.

**Conclusions:**

Included studies showed moderate risk of bias, inconsistent metrics, small cohorts, and limited dataset transparency. LLM-based virtual patient systems excel in simulating multiple disease types but lack multimorbidity patient representation. KGs improve top-k accuracy and support structured disease representation and reasoning. Future research should prioritize hybrid KG-chain-of-thought architectures integrated with open-source KGs (eg, UMLS [Unified Medical Language System] and SNOMED-CT [Systematized Nomenclature of Medicine - Clinical Terms]), parameter-efficient fine-tuning, dialogue compression, multimodal LLMs, standardized metrics, larger cohorts, and open-access multimodal datasets to further enhance realism, diagnostic accuracy, fairness, and educational utility.

## Introduction

Since 2020, large language models (LLMs) such as GPT-3.5 (OpenAI) [1] and GPT-4 (OpenAI) [2] have significantly enhanced virtual patient systems in medical education. Unlike traditional methods relying on resource-intensive standardized patients or high-fidelity simulators [3], LLMs provide scalable, low-risk, and cost-effective solutions by simulating realistic patient interactions across a wide range of clinical scenarios, including internal medicine, mental health disorders, and surgical and orthopedic cases [4]. This capability addresses key challenges in medical education, such as limited exposure to diverse clinical cases and the high costs of traditional simulation approaches.

Early virtual patient systems, often based on models like BERT (Bidirectional Encoder Representations from Transformers), struggled to generate natural dialogues and adapt to complex clinical scenarios, limiting their effectiveness in medical training [5,6]. In contrast, modern LLMs, leveraging prompt-based techniques for role and scenario customization, demonstrate improved contextual understanding, enabling clinically relevant responses. However, these systems still face challenges with hallucination, defined as the generation of factually incorrect or contextually irrelevant content, which may compromise the accuracy of medical history-taking in virtual patient simulations [7,8].

To ensure effective clinical training, continuous improvement and validation of LLMs are essential to mitigate hallucination rate and ensure the reliability of generated information [7]. Recent studies have incorporated techniques such as Supervised Fine-Tuning (SFT) [9] and Retrieval-Augmented Generation (RAG) [10] to enhance contextual adaptability and diagnostic accuracy, as measured by metrics like top-k accuracy and GTPA@k. However, the specific disease types simulated by LLMs, such as such as neurological and rheumatological or rare multiple disease types and potential gaps in simulation capabilities remain underexplored partly due to variations in experimental design and datasets.

Despite progress, systematic comparative evaluations of these techniques in virtual patient systems are lacking. Existing literature reviews often broadly discuss LLMs in medical education without focusing on virtual patient history-taking, limiting insights into domain-specific challenges [11]. Thus, a systematic literature review is critical to consolidate fragmented research, identify challenges, and guide future work.

Previous systematic reviews have explored LLMs in medical education but lack specificity. For instance, Lucas et al [11] reviewed LLMs’ implications for teaching effectiveness, ethics, and reliability but did not focus on virtual patient history-taking. García-Torres et al [12] used a hybrid human-LLM methodology to evaluate virtual patients’ impact on clinical reasoning but provided limited technical details on prompt design, knowledge graph (KG) integration, or fine-tuning. Similarly, Fatima et al [13] conducted a cross-disciplinary review of ChatGPT in research, clinical practice, education, and patient interaction but did not systematically analyze virtual patient history-taking. Recent empirical studies on LLM-powered virtual patients with automated feedback have prioritized educational outcomes over methodological comparisons [8].

In contrast, this review specifically examines LLM-based virtual patient systems for clinical history-taking. It systematically analyzes prompt engineering, external knowledge integration, model fine-tuning, and evaluation strategies, synthesizing their implications for medical education and effective clinical training. By adopting this methodological focus, this work addresses gaps in prior reviews, which were either too broad or lacked technical depth.

**Figure 1:**
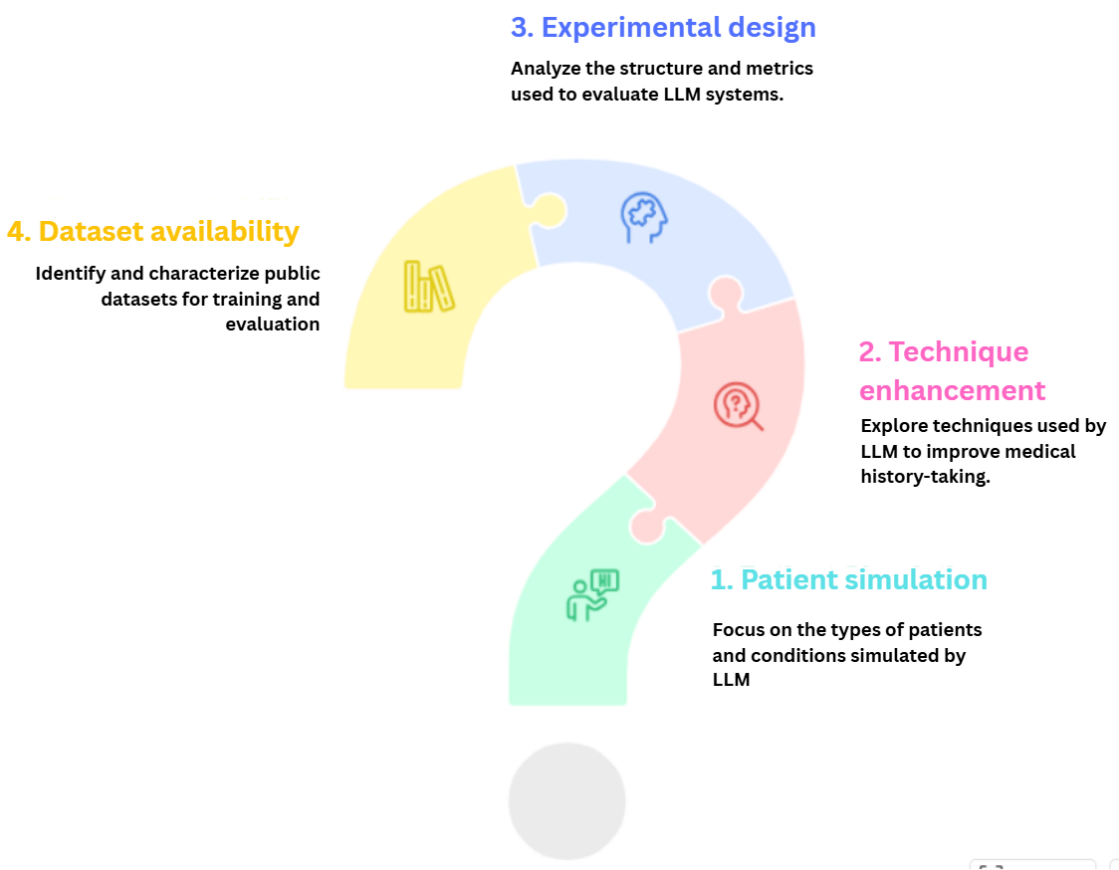
Overview of 4 research questions on large language model–based virtual patient systems, covering simulation types, enhancement techniques, evaluation strategies, and datasets.

Given this research gap, a systematic literature review is essential to consolidate fragmented research and guide future studies. This paper addresses four core research questions (RQs), as outlined in Figure 1:

- RQ1: what types of patients, conditions, or diseases, such as internal medicine or rare and multiple disease types, are simulated in LLM-based virtual patient systems?
- RQ2: what techniques do LLMs use to enhance medical history-taking capabilities in clinical interviews?
- RQ3: how are experimental designs structured to evaluate LLM-based virtual patient systems, and what evaluation metrics, such as top-k accuracy or System Usability Scale (SUS), are used?
- RQ4: what public datasets are available, and what are their characteristics for training, simulating, and evaluating medical history–taking in virtual patient systems?

This systematic literature review addresses these questions to guide researchers in understanding the potential, limitations, and ethical challenges of LLMs in virtual patient systems. It is critical for advancing effective clinical training tools in medical education. The paper is structured as follows, the first section introduces the background of virtual patient systems and LLM applications; the second section describes the literature search methodology and evaluation criteria; the third section presents results, analyzing effectiveness and challenges; the fourth section discusses findings in the context of existing literature; and the fifth section concludes with key findings and recommendations for future research.

## Methods

### Overview

This study conducted a systematic literature review adhering to PRISMA (Preferred Reporting Items for Systematic Reviews and Meta-Analyses) 2020 guidelines. The research topic and target population were clearly defined, a comprehensive search strategy was formulated, and key search terms were identified. Search results were exported as CSV files via Mendeley (Mendeley Ltd) and Zotero (Corporation for Digital Scholarship) and manually screened. Abstracts were initially evaluated, followed by a comprehensive full-text assessment to confirm relevance and quality.

### Eligibility Criteria

#### Population

The population includes medical students and physicians engaged in medical history–taking. Artificial intelligence (AI)–based virtual patients refer to LLM-based systems simulating patients for training in history-taking. Only transformer-based LLMs (eg, GPT series) are included, excluding older models like BERT (Google AI) or pre-Transformer architectures. Studies focusing on anesthesia, emergency procedures, or intraoperative scenarios are excluded, as these are nontraditional history-taking contexts. Preoperative history-taking for surgical and orthopedic patients is included. This ensures a focus on transformer-based LLMs for history-taking, emphasizing educational and clinical skill development while excluding irrelevant clinical settings. Only studies where LLMs serve as virtual patients for active history-taking and patient communication are included, excluding those focused solely on medical interviews without history-taking.

#### Intervention

The intervention involves transformer-based LLM technologies applied to medical history-taking, including prompt design, model fine-tuning, KG integration, and other LLM-related techniques. These aim to enhance diagnostic accuracy, interaction quality, and overall performance in virtual patient systems.

#### Outcomes

Outcomes include performance metrics such as top-k accuracy (eg, top-1 accuracy), empathy scores, readability, system stability (eg, response consistency), user experience (eg, SUS, Chatbot Usability Questionnaire [CUQ]), and other relevant indicators, such as *κ* (Cohen κ) and *P* value. These metrics evaluate the effectiveness of transformer-based LLM virtual patient systems in medical history–taking.

#### Inclusion and Exclusion Criteria

Inclusion and exclusion criteria were established to ensure relevance and quality, as shown in Textbox 1. Inclusion criteria prioritize studies with technical depth, validated outcomes, recent publication, and a focus on Transformer-based LLM virtual patients for history-taking, excluding nontransformer models.

**Inclusion and exclusion criteria for transformer-based large language model (LLM) virtual patient studies.**

**Inclusion criteria:**

- IC1: the study population included medical students or physicians involved in medical history–taking or communication training.
- IC2: the intervention involved Transformer-based LLMs (eg, GPT series) used as virtual patients for history-taking or communication training in traditional consultation settings.
- IC3: the study was published between January 1, 2020, and August 18, 2025, including peer-reviewed articles and preprints.
- IC4: the study reported measurable outcomes related to diagnostic performance, communication effectiveness, empathy, readability, or user experience.
- IC5: preoperative history-taking scenarios (eg, surgical or orthopedic patients) were included if they involved conventional patient–clinician consultation processes.

**Exclusion Criteria:**

- EC1: based on earlier or nongenerative transformer models (eg, BERT [Bidirectional Encoder Representations from Transformers] and GPT-2) rather than modern LLMs.
- EC2: focused solely on medical interviews without explicit medical history–taking.
- EC3: published before January 2020.
- EC4: duplicate titles or redundant publications.
- EC5: review or commentary papers.
- EC6: non-English language studies.
- EC7: focused on anesthesia, emergency procedures, or intraoperative contexts (except preoperative consultations).

### Information Sources

A systematic literature search was conducted across 9 authoritative databases PubMed, Scopus, Web of Science, IEEE Xplore, ACM Digital Library, SpringerLink, ERIC, arXiv, and ACL Anthology, covering studies published between January 2020 and August 18, 2025, in medicine, AI, education, and virtual technologies. A secondary search used a snowballing strategy, identifying additional sources from 15 initial articles and relevant publications, including theses from ProQuest. Including peer-reviewed articles and grey literature (eg, preprints from arXiv and theses) ensured comprehensive coverage of the rapidly evolving fields of AI and medical education.

### Search Strategy

Two search strategies were used for this systematic review. The first query combined virtual patient terms (eg, virtual patient, simulated patient, AI patient, conversational patient, chatbot patient, intelligent virtual agent, and dialogue agent) with LLM-related keywords (eg, LLM, ChatGPT, GPT-4, GPT, transformer model, generative AI, AI-powered tutor, and natural language generation) and was applied to structured databases including PubMed, Scopus, Web of Science, IEEE Xplore, and ACM Digital Library. The second, simplified query included only virtual patient terms and was applied to broader repositories, namely SpringerLink, ERIC, arXiv, and ACL Anthology, to ensure comprehensive coverage, as strict LLM keywords reduced results in these databases (see Multimedia Appendix 1 for more details).

### Selection Process and Data Collection

The literature screening and data collection process followed PRISMA 2020 guidelines [14] (checklist provided in Multimedia Appendix 2). Titles and abstracts were manually screened for relevance to LLM-based virtual patient history-taking, with GPT-3.5 Turbo used for auxiliary verification. Full-text evaluations were conducted for studies meeting preliminary inclusion criteria. A multidimensional quality assessment form recorded key study characteristics and outcomes, as detailed in Multimedia Appendices 3 and 4. The ChatPDF tool was used to cross-check content, and final inclusion decisions were made with the supervising researcher’s input.

### Quality and Risk of Bias Assessment

A reviewer with a computer science background, under the supervision of an experienced research advisor, conducted the quality assessment. A customized multidimensional assessment framework was developed to evaluate the technical quality of the included studies, as described in Multimedia Appendices 3 and 4. Conventional appraisal tools such as the Joanna Briggs Institute Critical Appraisal Checklist (2020) were adapted because they were not fully suitable for LLM-based virtual patient research. The framework incorporated 6 evaluation dimensions methodology clarity, dataset transparency, completeness of system evaluation, innovation or integration level, reproducibility and openness, and the presence of control or baseline comparisons. Each dimension was rated on a 3-point scale ranging from 0 to 2, with 2 indicating the highest quality. The total score for each study, therefore, ranged from 0 to 12. Based on the overall score, studies were categorized into 3 quality levels, high (9-12 points), medium (5-8 points), and low (0-4 points). This classification ensured a consistent and transparent interpretation of the technical quality across studies. The assessment emphasized methodological rigor, technical implementation, system architecture, model training strategies, multimodal integration, and evaluation methods to identify potential sources of bias.

Risk of bias was also assessed across 5 domains—selection or reporting bias, implementation bias, evaluation bias, data bias, and reporting completeness bias—to capture variations in study design, data transparency, technical implementation, and reporting quality. This combined approach ensured a systematic evaluation of both methodological soundness and potential bias in LLM-based virtual patient studies.

Based on the aforementioned screening and evaluation methods, the research results will be presented in detail in the next section.

## Results

### Overview

This section reports the results of literature selection, study characteristics, and findings related to the 4 RQs, avoiding interpretive discussion.

### Study Selection Results

**Figure 2:**
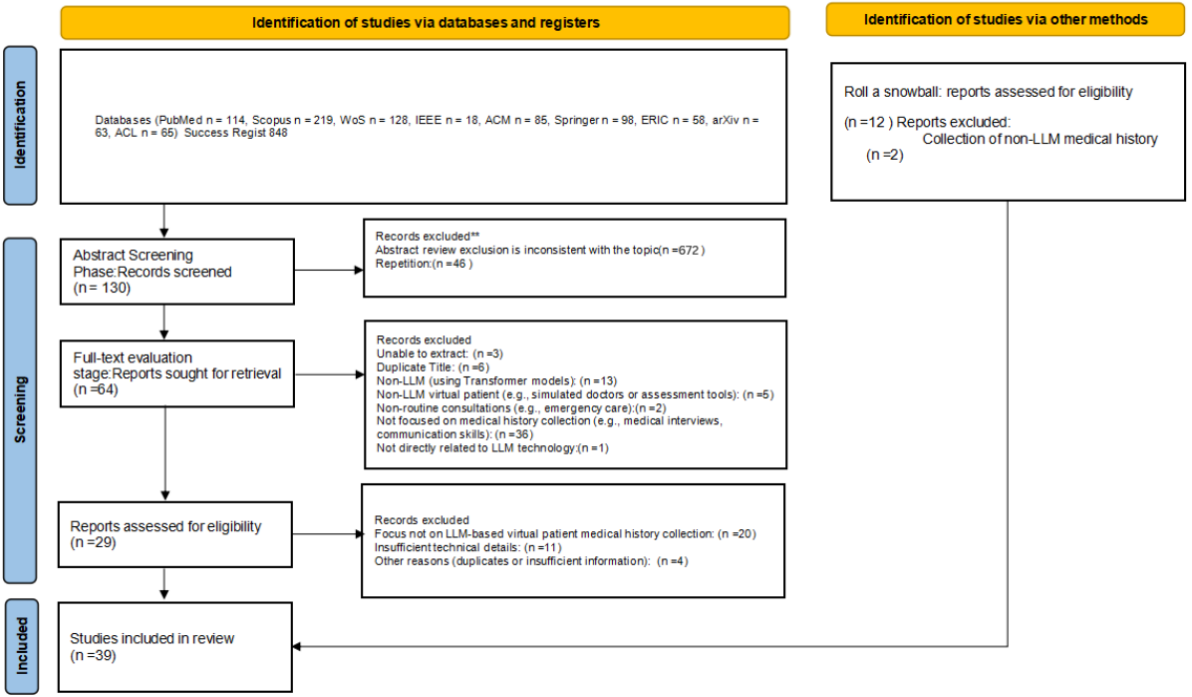
PRISMA flow diagram.

During identification (see Figure 2 for the 2020 flow diagram for systematic reviews, including database and register searches), 848 records were retrieved from 10 databases. During abstract screening, 672 records were excluded, and after removing 46 duplicates, 130 unique full-text articles were assessed. Of these, 66 were excluded for reasons including lack of extractable data (n=3), duplicate content (n=6), non-LLM models (n=13), focus not on LLM applications (n=5), nonroutine clinical settings (n=2), no history-taking focus (n=36), or nonrelevance to LLM technologies (n=1), leaving 64 eligible articles. During multidimensional evaluation, 20 studies were excluded for limited relevance, 11 for insufficient technical detail, and 4 for other reasons, yielding 29 articles from database searches. An additional 12 articles were identified through snowballing, of which 2 were excluded as irrelevant, resulting in 10 included snowballed studies. The final synthesis comprised a total of 39 studies [8,10,15-51] (6 high-quality, 33 moderate-quality) (see Multimedia Appendix 5).

### Quality and Risk of Bias Assessment Results

Most studies achieved the highest rating (2 points) for methodological clarity, indicating well-defined research procedures and consistent experimental designs. Scores for dataset transparency and system evaluation completeness varied considerably, with approximately 45%-55% of studies receiving moderate ratings (1 point) due to insufficient details regarding data sources or evaluation frameworks. Innovation and integration levels were generally high, with about 80% of studies receiving high ratings (2 points), reflecting notable progress in multimodal integration and technological creativity. Reproducibility and openness received moderate ratings (1 point) in approximately 60% of studies, as some provided replication details whereas others lacked information on model configurations or training strategies. Control or baseline comparison obtained the lowest scores (0-1 point), with only 25%-30% of studies incorporating explicit comparative or controlled analyses. Overall, 6/39 (15%) studies were rated as “high quality,” and 33/39 (85%) as “moderate quality,” suggesting strong methodological design and innovation but highlighting the need for improved dataset transparency, evaluation completeness, and reproducibility (see Multimedia Appendix 5 for details).

**Figure 3:**
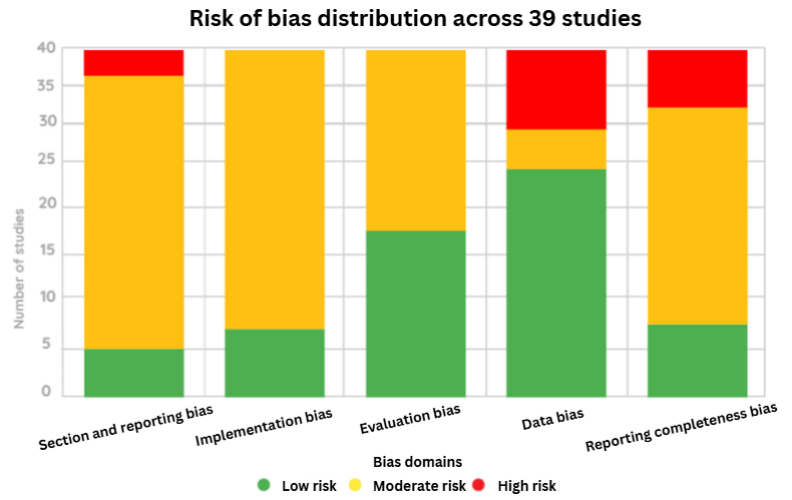
Overview of risk of bias distribution across studies.

Risk of bias was assessed across five domains—selection and reporting bias, implementation bias, evaluation bias, data bias, and reporting completeness bias—as illustrated in Figure 3.

Selection and reporting bias was evident in disease coverage. Approximately 95% (37/39) of studies simulated specific disease types (eg, internal medicine or mental health disorders), but only 50% (20/39) addressed multiple disease stages, thereby limiting generalizability to multimorbidity contexts.

Implementation bias was observed in the limited reporting of model development details. While 80% (31/39) of studies described model types, only 35% (14/39) provided information on training procedures, fine-tuning processes, prompt engineering, reinforcement learning with human feedback (RLHF), domain-specific adaptation, or multimodal integration. This resulted in high technical heterogeneity.

Evaluation bias was reflected in the uneven assessment of educational and user experience outcomes. Approximately 70% (27/39) of studies evaluated educational outcomes, and 65% (25/39) assessed user experience using instruments such as the SUS or the Chatbot Usability Questionnaire (CUQ). However, only 30% (12/39) of studies conducted comparative or controlled evaluations, which limited the interpretability of effectiveness findings.

Data bias stemmed from moderate dataset transparency. Fewer than half of the studies (18/39, 45%) explicitly identified data sources or quality control procedures, constraining reproducibility.

Reporting completeness bias was identified in the limited documentation of personalization mechanisms and quality control for generated outputs, reported in only 25%-30% (10-12/39) of studies, suggesting incomplete methodological reporting.

In summary, this multidimensional quality and bias assessment systematically examined the technical features, methodological design, and potential biases of LLM-based virtual patient research. The findings highlight the need to improve dataset transparency, enhance the comprehensiveness of system evaluation, and include controlled comparative studies to strengthen reproducibility and scientific validity. Details of the screening results and full bias assessment are provided in Multimedia Appendix 6.

### Finding for RQ1: What Types of Patients Are Simulated in LLM-Based Virtual Patient Systems?

This section presents a descriptive statistical analysis and classification of patient types simulated by LLMs in virtual patient history-taking systems, based on a systematic literature review. The classification uses disease categories from Table 1, grouping similar disease types (eg, internal medicine and mental health disorders). For each category, the number of studies, specific simulation scenarios, disease complexity (eg, low: single symptom; medium: multisystem involvement; high: rare or complex interactions), number of simulated cases (if specified), and disease stage (eg, acute and chronic) are summarized.

**Table 1:** Classification of patient types simulated in virtual patient systems.

Disease category	Simulation scenarios	Complexity	Number of cases	Stage	References
Internal medicine	Chest/abdominal pain, diabetes, COPD^a^, COVID-19, hypertension, multisymptom	Medium-high	3-500+	Acute-chronic	12 studies [15,20-30]
Mental health disorders	Depression, PTSD^b^, ADHD^c^, TRD^d^, CBT^e^ models	High	1-106	Acute-chronic	8 studies [18,31-35,37]
Surgical/orthopedic	Plastic surgery, hand surgery, joint pain	Medium	3-10	Acute–chronic	4 studies [16,20,24,38]
Neurological/rheumatological	Stroke, meningitis, concussion, polymyositis, brain hemorrhage	High	1-4	Acute	6 studies [20,21,36,39-41]
Ophthalmological	Eye conditions, pain with redness/photophobia	Medium	1-24	Acute	3 studies [17,20,42]
Dermatological	Ear cyst, telogen effluvium, skin conditions	Medium	1-394	Acute-chronic	4 studies [19,25,43,44]
Rare/multiple disease types^f^	Rare diseases, unspecified conditions, broad patient scenarios	High	8-5230	Diverse	10 studies [8,10,25,45-51]

^a^COPD: chronic obstructive pulmonary disease.

^b^PTSD: posttraumatic stress disorder.

^c^ADHD: attention deficit hyperactivity disorder.

^d^TRD: treatment-resistant depression.

^e^CBT: cognitive behavioral therapy.

^f^Only [25] involves rare disease simulation, using the RareBench dataset with 421 rare disease cases. Other references in the “rare/multiple disease types” category focus on multiple disease types or unspecified/broad patient scenarios.

### Summary of Research Focus and Coverage

A systematic descriptive analysis of the literature indicates that research primarily focuses on internal medicine, particularly gastrointestinal (eg, abdominal pain, heartburn, hematemesis [20-23,27,28]), respiratory (eg, cough, chronic obstructive pulmonary disease, COVID [20,21,23,29,30]), cardiovascular (eg, chest pain, hypertension [20-22,30]), metabolic and endocrine (eg, diabetes [10,23,24]), and fatigue-related conditions (eg, chronic fatigue [15,26]). These conditions are well-suited for effective clinical training, with LLMs effectively simulating both acute and chronic management scenarios, though vague symptoms like fatigue remain underexplored.

Mental health disorders are a significant focus, including depression and related disorders (eg, suicidal ideation and treatment-resistant depression) [15,32-35], posttraumatic stress disorder (posttraumatic stress disorder; eg, combat trauma) [18,31], attention-deficit/hyperactivity disorder [31], and other mental health conditions (eg, cognitive impairment, cognitive behavioral therapy [CBT] models) [32,37]. These simulations emphasize emotional and psychological complexity, suitable for empathy and CBT training, though behavioral disorders like attention-deficit/hyperactivity disorder are less explored.

Rare and multiple disease types are well represented, focusing on heterogeneous case collections and electronic health record (EHR)–driven simulations [8,46-51], as well as rare disease modeling (eg, 421 rare diseases [10,25,45]). These studies highlight the scalability and diversity of LLMs in simulating complex and varied clinical conditions.

Less studied areas include neurological and rheumatological diseases (eg, stroke, meningitis, and polymyositis [20,21,36,39-41]), dermatological diseases (eg, ear cyst, telogen effluvium, and skin conditions [19,25,43,44]), surgical and orthopedic diseases (eg, joint pain, plastic surgery, and hand surgery [16,20,24,38]), and ophthalmological diseases (eg, eye pain, photophobia, and multiple eye conditions [17,20,42]). These areas, due to specialized or visual simulation requirements, have received less attention, revealing significant research gaps.

Overall, research focuses on internal medicine and mental health disorders due to their prevalence and clinical relevance. Rare and multiple disease types demonstrate LLMs’ scalability and generalization potential. In contrast, specialized domains such as surgical and orthopedic, neurological and rheumatological, dermatological, and ophthalmological diseases remain underexplored, presenting opportunities for further innovation in simulation-based medical education.

### Finding for RQ2: What Techniques do LLMs Use to Enhance Medical History–Taking Capabilities in Clinical Interviews?

To address RQ2, LLMs use advanced techniques to enable realistic, dynamic, and accurate virtual patient simulations for medical history-taking, providing medical students and professionals with effective clinical training platforms that closely resemble real-world scenarios. The following sections categorize core LLM technologies for history-taking into prompt engineering, KGs and structured data, model fine-tuning and training, and speech interaction, as summarized in Table 2.

**Table 2:** Technical metrics, evaluation scores, and key strengths and limitations of LLM virtual patient techniques.

Category and technique (source)^a^			Top-k accuracy (%)		Hallucination rate (%)		IRR^b^ (%)		AS^c^ (%)		Strengths		Limitations
**Prompt design**													
	Role-based prompts [10,23]	81.4		4.97		2.08		28		Realistic role-play; multiagent;		Long prompts inconsistent; complex cases limited	
	Few-shot [25]	Not used: 25%, used: 52%								Simple, effective;		Limited depth	
	Multiagent prompt frameworks [21,24]	—^d^		—		—		—		Consistent dialogue		Higher computation	
**KG^e^**													
	Entity-relation triples [17]	—		—		—		—		Structured triples		Small-scale realism	
	Entity layering [10]	97.42		—		—		—		High diagnostic accuracy and interpretability		Limited semantic layers	
	KG+history and few-shot [10]	97.85		—		—		—		Role continuity		Context overflow	
	KG+multiagent prompts [10]	97.85		—		—		—		Modular roles		High cost	
**Fine-tuning**													
	SFT^f^ [17]	—		(Before fine-tuning 3.71%, after fine-tuning 0.31%)		4.79		87.00		Reduces hallucination rate; better reasoning		Limited turn validation	
	LoRA^g^ [17]	—		—		—		—		Efficient tuning		Limited scalability evidence	
**Speech interaction**													
	TTS^h^ [28,51]	—		—		—		—		Realistic voice; multimodal input		Recognition errors possible	

^a^All techniques listed are LLM-based Transformer architectures.

^b^IRR: information-related response rate.

^c^AS: Anthropomorphism Score.

^d^Not available.

^e^KG: knowledge graph.

^f^SFT: supervised fine-tuning.

^g^LoRA: low-rank adaptation.

^h^TTS: text-to-speech.

#### Prompt Design for Realistic Patient Simulation

1. Role-based prompt design: detailed prompts embed patient demographics, personality traits (eg, Big Five), and clinical symptoms to simulate authentic patient behavior. For instance, Borg et al [41] developed virtual patient cases with approximately 2000 tokens per prompt, including detailed medical histories and contextual information, occupying a significant portion of the LLM’s 4096-token context window. Implementations include structured prompts with role context, medical history, and behavioral constraints [16-20,22,24-29,31,33-41,43-46,51]. For example, Bodonhelyi et al [37] used Satir model roles (eg, “accuser”') to achieve up to 95% dialogue consistency. This ensures consistent role-playing but requires instructor oversight to maintain medical accuracy [36].
2. Few-shot and dialogue history–based prompting: prompts incorporate dialogue history and few-shot examples to maintain conversational coherence and mimic gradual information disclosure. MEDDxAgent uses structured examples to guide diagnostic reasoning, improving top-k accuracy from 25% to 52% in complex cases [25]. Limitations include token constraints, which may restrict handling of intricate scenarios.
3. Multiagent prompt frameworks: multiple agents (eg, patient agent, doctor agent, and behavior controller) collaborate to generate realistic and personalized clinical dialogues, reducing reliance on single prompts and minimizing hallucination rate. For example, EvoPatient, developed by Du et al [18], uses a patient–doctor dual-agent system for natural dialogue via unsupervised learning. LLM-based generative agents integrate memory flows, retrievers, and cognitive mechanisms to enhance dialogue realism and training effectiveness [24]. Additionally, AI self-play agents such as AMIE simulate diagnostic conversations through internal and external self-play loops, applying multirole strategies within a single model to improve diversity and dialogue adaptation [21].

#### Knowledge Graphs and Structured Data

1. Dynamic KG retrieval: MedDiT uses KG agents to retrieve relevant subgraphs via SPARQL queries, linearizing them into natural language prompts to reduce token load and context loss, achieving significant token reduction [47].
2. Hierarchical KG modeling: AI Patient integrates layered KG with a Reasoning RAG multiagent framework, boosting accuracy from 68.94% to 94.15%. For the difficult “family and social history” category, accuracy improved from 13.33% to 85.56%, showing the value of structured KG reasoning. Compared to role-based prompts, the KG-based method performs better in multiclass tasks. With entity layering [10], accuracy rose by 16.02% (81.4% to 97.42%) in challenging categories such as allergy and social history.

#### Model Fine-Tuning and Training

1. Instruction fine-tuning and self-play: AMIE uses real and simulated medical dialogues with self-play loops (inner and outer) to optimize diagnostic performance, outperforming primary care physicians (PCPs) [19,45].
2. SFT and LoRA (Low-Rank Adaptation): Liu et al [17] fine-tuned Qwen2.5-72B-Instruct on MedDialog, reducing the hallucination rate from 3.71% to 0.31% [17]. At the same time, in terms of information-related response rate and Anthropomorphism Score (AS).
3. Chain-of-thought (CoT) and RLAIF+MoM: CureFun uses CoT and RAG, while Kumar et al’s [26] RLAIF+MoM framework structures ambiguous symptoms, achieving up to 95% output clarity [23].

#### Speech Interaction

1. TTS and STT integration: Takata et al [28] use Google application programming interface (API) and Unity for synchronized speech [51] and emotional facial expressions, outperforming traditional platforms in interaction realism. Thesen et al [51] leverage Whisper-3 for immersive speech processing, achieving high student satisfaction.
2. Multimodal enhancements: AMIE integrates image uploads (eg, skin photos) to support history-taking, outperforming PCPs in diagnostic accuracy [19]. Ryu et al [35] enhances realism with patient image uploads.

In conclusion, LLM technologies achieve consistent and realistic virtual patient dialogues through multilevel prompt design, few-shot examples, and multiagent mechanisms. The use of KGs and structured data enhances information retrieval and medical history modeling, improving classification and recognition accuracy. Model fine-tuning techniques (SFT, LoRA, self-play, CoT, RLAIF+MoM) effectively reduce hallucination rate, optimize reasoning, and enhance diagnostic performance. Furthermore, speech interaction and multimodal integration improve interaction immersion and provide diagnostic support.

### Findings for RQ3: How Are Experimental Designs Structured to Evaluate LLM-Based Virtual Patient Systems, and What Evaluation Metrics Are Used?

To address RQ3, this review synthesized 32 studies listed in Table 3 to examine the experimental design, evaluation methods, and metrics of LLM-based virtual patient systems, focusing on medical education, high diagnostic accuracy, and effective clinical training. Of the 39 identified studies (see Multimedia Appendix 5), 7 were excluded due to the absence of formal evaluations (eg, Takata et al [28] described planned behavioral experiments without data collection; Rodrigo et al [46] outlined testing plans but reported no results; Geer [31] focused on design without providing quantitative outcomes; Li et al [47] emphasized system architecture but lacked performance metrics; Staples et al [34] relied solely on qualitative feedback; Kumar et al [26] discussed workflow but lacked quantified results; Lee et al [15] provided expert Likert scores without statistical analysis). Due to high heterogeneity in evaluation metrics (eg, top-k accuracy, hallucination rate, CUQ, inconsistent scales and dimensions, lack of statistical information such as SDs or confidence intervals in many studies, and large variations in sample sizes that could bias pooled analyses), a meta-analysis was not performed. Instead, a structured narrative synthesis was adopted, supplemented with tabular summaries in Tables 2 and 3, which present the technical approaches and corresponding evaluation metrics.

**Table 3:** Merged quantitative and qualitative evaluation details for virtual patient systems.

Authors	Participants (N)	Identity	Comparisons	Analysis method	Results	Evaluation type
Du et al [18]	None	AI^a^	Evolved vs Nonevolved	Stats^b^	Relevance 0.7589; Faithfulness 0.8786	Q^c^
Holderried et al [8]	106	Med students	GPT-4 vs Human	*κ* ^d^	Top-k accuracy >99%;*κ*=0.832	Q
Tu et al [21]	20	PCPs^e^	AMIE vs PCPs	Stats	AMIE better on 28/32 expert metrics	Q
Yamamoto et al [20]	145	Med students	AI vs Traditional	t and MWU^f^; Likert^g^	OSCE: 28.1 vs 27.1 (*P*^h^=.01)	M^i^
Cook et al [50]	3	Physicians	GPT-4.0 vs 3.5	1–6 scale, multivar.	GPT-4.0 higher authenticity, feedback	Q
Haider et al [16]	None	AI	GPT-4o vs Claude vs Gemini	Stats	Nonsignificant differences; high baseline	Q
Brugge et al [39]	21	Med students	Feedback vs Control	Wilcoxon, ICC^j^=0.924	CRI-HTI: 3.60 vs 3.02 (*P*^k^≈.05)	Q
Holderried et al [22]	28	Med students	None	Stats; Spearman	Script Q 60.3%; Answers 94.4%	Q
Leypold et al [38]	3	Hand surgeons	None	Likert (1–5)	Mean=4.6 (Understand 5.0; History 4.2)	Q
Borg et al [40]	15	Med students	Robot vs VP	*t* test; Thematic	Authenticity 4.5 vs 3.9 (*P*^l^=.04)	M
Radel et al [36]	40	Med students	Feedback vs None	*t* test; Likert	Improved scores with feedback (*P*^m^<.05)	M
Luo et al [42]	184	Med students	LLMDP vs Traditional	*t* test; Pearson	78.13 vs 67.08 (*P*^n^<.001)	M
Thesen et al [51]	94	Med students	None	Likert; *t* test	Comfort 61% to 76% (*P*^o^<.001)	M
Laverde et al [24]	86	Med students	Agent vs Others	CUQ^p^	CUQ: 86.25/100	Q
Benfatah et al [29]	12	Nursing students	None	Pearson; Likert	Total score 19.42; Clarity r=0.701	M
Geer [31]	None	AI	None	Descr^q^	High similarity; no quant data	QL^r^
Borg et al [41]	15	Med students	Robot vs VIC	Wilcoxon; Text	Authenticity 4.47 vs 3.93 (*P*^s^≈.03)	M
Ng et al [49]	100	Med students	Hybrid vs Baselines	Acc^t^; failure and confusion	Top-k accuracy 98.7%; failure 2.0%	Q
Wang et al [32]	N/S	Experts	Expert vs GPT-4	Stats; Subjective	Experts rated higher, GPT-4 underestimated	QL
Zheng et al [48]	N/S	Experts	None	Weighted *F*_1_-score; Fuzzy labels	High professionalism and ethics	M
Rose et al [25]	None	AI	GPT-4o vs Llama3.1	Stats	GTPA@1 0.96; RareBench 0.45	Q
Liu et al [17]	None	AI	Proposed vs Baselines	Stats	Hallucination Rate 0.31%; Anthropomorphism 0.87	Q
Chen et al [33]	25	Patients and Psychiatrists	Prompt D1–D4	Stats	Fluency 3.28; Empathy 3.43; Dx 55.56%	Q
Liao et al [43]	N/S	Students, laypeople	GPT-4 vs others	Stats	Dx 53.33%; Coverage rate:15.36%-33.89%%	Q
Johri et al [44]	None	AI	GPT-4 vs 3.5	Stats	MCQ 0.919; FRQ 0.684	Q
Bodonhelyi et al [37]	N/S	Psych experts	Accuser vs Rationalizer	Likert; Emotion; Stats	Realism 3.8 vs 3.7	M
Rashidian et al [30]	2	Clinicians	AI vs Doctors	*κ*; Likert	Symptom top-k accuracy 97.7%; *κ*=0.74	M
Tu et al [45]	20	PCPs	AMIE vs PCPs	Stats	AMIE better on 28/32 expert metrics	Q
Saab et al [19]	43	Patients, specialists	AMIE vs PCPs	Stats; *P* values	*Top*-1 accuracy: 0.65 vs 0.53 (*P*^u^<.001)	Q
Li et al [23]	8	Med experts	Auto vs Manual	Spearman; Pearson	*ρ*=0.81; *r*=0.85 (*P*^v^<.05)	Q

^a^AI: artificial intelligence.

^b^Stats: statistical analysis.

^c^Q: Quantitative.

^d^*κ*: Cohen κ.

^e^PCP: primary care physician.

^f^t and MWU: *t* test/Mann-Whitney *U* test.

^g^Likert: Likert scale.

^h^*P*: Objective Structured Clinical Examination performance comparison between AI-trained and traditionally trained students showed a difference of 28.1 vs 27.1, respectively (Mann–Whitney *U* test, *P*=.01).

^i^M: Mixed.

^j^ICC: intraclass correlation coefficient.

^k^*P*: Feedback group scored 3.60 on CRI-HTI vs 3.02 in control group (ANOVA, ICC=0.924, *P*=.049).

^l^*P*: Robot vs virtual patient authenticity ratings were 4.5 vs 3.9 (*t* test; *P*=.04).

^m^*P*: Students receiving feedback showed higher Likert-scale scores compared to control (*t* test; *P*=.04).

^n^*P*: LLMDP-trained students scored 78.13 (SD 8.35) on history acquisition; traditional group scored 67.08 (SD 7.21), with a mean difference of 11.05 points (*P*<.001).

^o^*P*: Comfort scores improved from 61% to 76% after intervention (*t* test; *P*<.001).

^p^CUQ: Chatbot Usability Questionnaire.

^q^Descr: Descriptive.

^r^QL: Qualitative.

^s^*P*: Robot vs VIC comparison yielded authenticity ratings of 4.47 vs 3.93 (Wilcoxon test; *P*=.035).

^t^Acc: Accuracy.

^u^*P*: Original article did not provide comparative statistical values such as means or test statistics, only reported model accuracy with significance levels.

^v^*P*: Among the 8 evaluated cases, exact *P* values for comparisons 1 and 2 were *P*<.001 and *P*=.04, respectively; the remaining 6 ranged from *P*=.003 to *P*=.011. As per reporting guidelines, *P*=.000 was converted to *P*<.001.

### Commonalities and Specificities in Experimental Design

#### Commonalities

Most studies involved medical or health professional students, typically 10-50 participants, with some including residents or practicing physicians as evaluators (usually 3-5 experts) [10,19,36]. A few studies used AI agents for large-scale automated testing [18,25]. Tasks primarily covered core clinical competencies, including history-taking accuracy [10,36], diagnostic reasoning [45], role-playing [37], and multiturn dialogues [41].

Evaluation paradigms were categorized into 3 types quantitative (18 studies) using randomized controlled trials (RCTs) or comparative experiments with statistical tests (eg, ANOVA, *t* tests, and correlation) [39,45]; qualitative (1 study) using expert interviews, thematic analysis, or questionnaires [32]; and mixed (13 studies) integrating objective metrics (eg, top-k accuracy, and *F*_1_-score) with subjective scales (eg, Likert, CUQ) [36,40]. Comparative baselines included model comparisons (GPT-4 and 4o, Claude, and Gemini) [16], AI versus human physicians [19,45], with or without feedback or platform comparisons [40,41], and variations in prompt strategies, multimodality, or RAG and KG [49]. Most studies applied blinding and reported rater consistency (eg, *κ*) [8,39]. Details of each study’s evaluation type are provided in Table 3 (Evaluation type column).

#### Specificities

Some studies implemented AI self-play for scalability [18], or AI doctor-AI patient automated evaluation [16]. Social robot-LLM hybrids were used to enhance realism [40,41]. Comparative baselines varied, including direct AI versus human physician comparisons [19,45], modular and hybrid architecture comparisons (eg, RASA or KG or LLM) [49], and multimodel comparisons (GPT-4.5 and 4o, Claude 3.7, and Gemini 2.5) showing nonsignificant differences, indicating a high performance baseline [16].

### Evaluation Metrics: Commonalities and Specificities

Evaluation metrics in LLM-based virtual patient systems generally fall into 5 categories: clinical accuracy and knowledge, communication and interaction quality, robustness and stability, training efficacy and feedback quality, and system performance. Clinical accuracy and knowledge were assessed using metrics such as top-k accuracy [19], GTPA@k [25], information coverage [43], hallucination rate [17,19], and fidelity and relevance [18], evaluating diagnostic and information-gathering capabilities. Communication and interaction quality were measured through readability (Flesch and Flesch–Kincaid) [10], CUQ [22], and Anthropomorphism Score [17,37], reflecting language clarity and interaction naturalness. Robustness and stability were evaluated via paraphrase robustness [10], leak resistance [18], and rater consistency metrics, including *κ* [8] and intraclass correlation coefficient (ICC) [39]. Training efficacy and feedback quality focused on learning outcomes and user experience, assessed through Objective Structured Clinical Examination (OSCE) score improvements [39], and usability scales (such as CUQ). System performance metrics, including latency, failure rate [49], and confusion or clarification rates [49], captured efficiency and reliability. Specificities included unique metrics such as span-level *F*_1_-score [10] for knowledge extraction, cosine similarity-based fidelity and relevance [18], weighted *F*_1_-score or fuzzy labels for professionalism and ethics [39], and system-level confusion, clarification, or failure rates. Automated evaluation metrics such as GTPA@k [25] provided standardized measures for diagnosis, while expert ratings revealed realism biases, including underestimation by GPT-4. Usability was further examined through instruments such as the CUQ (score of 77/100), 7-dimensional Likert scales combined with ANOVA for accuracy, realism, and empathy, and system latency and confusion reporting (0.5%). These metrics collectively enable comprehensive assessment of both the technical performance and experiential quality of LLM-based virtual patient systems. The calculation formula for the indicators is detailed in Multimedia Appendix 7.

### Evaluation Results

The synthesis of evaluation results from the 32 included studies indicates that LLM-based virtual patient systems demonstrate high diagnostic accuracy across key metrics. Quantitative results showed top-k accuracy ranging from 0.45 to 0.98 (based on 34 models and 300 patient cases); Saab et al [19] reported that AMIE achieved a Top-1 Accuracy of 0.65, compared to 0.53 for PCPs, with *P*<.001. Information coverage averaged 33.89% to 94.4% (eg, Liao et al [43] reported 33.89%, based on 150 cases; Holderried et al [22] reported 94.4%, based on 502 questions explicitly covered in case scripts, with a total of 826 answers), while hallucination rate remained low, ranging from 0.31% to 5% (eg, Liu et al [17] conducted experiments with up to 5 dialogue rounds, achieving a hallucination rate of 0.31%).

In terms of communication quality, CUQ scores ranging from 77 to 86.25 (eg, Laverde et al [24] reported 86.25). Training effectiveness was evident in OSCE performance (eg, Yamamoto et al [20] reported posttraining 28.1 vs pretraining 27.1, *P*=.01; Luo et al [42] reported that medical students trained using the LLM-based digital patient system (LLMDP) achieved a medical history acquisition score of 78.13 (SD 8.35), while the control group trained with traditional real patients scored 67.08 (SD 7.21). The difference between groups was 11.05 points, with *P*<.001, indicating a highly statistically significant difference). Robustness metrics such as *κ* ranged from 0.74 to 0.832 (eg, Holderried et al [8] reported 0.832; Rashidian et al [30] reported 0.74).

Mixed methods studies reported improvements in authenticity (eg, Borg et al [40] reported postuse 4.5 vs preuse 3.9; *P*=.04) and comfort (Thesen et al [51] reported that among 69 participants, average preuse comfort was 61%, increasing to 76% postuse, indicating higher student-reported comfort after training; *P*<.001). Automated baseline systems, such as EvoPatient, achieved a relevance of 0.7589 and a faithfulness of 0.8786 (Du et al [18]), while multimodel comparisons showed no significant differences among top LLMs (Haider et al [16]).

These results highlight the effectiveness of LLM-based systems in simulating realistic interactions, although specific metrics—such as the low failure rate of 2.0% reported by Ng et al [49] (based on 200 dialogue trials) and the high ICC of 0.924 reported by Brugge et al [39]—indicate that further standardization is needed.

The reviewed studies exhibit consistent commonalities in participant composition, task objectives, and evaluation paradigms, typically involving small to medium student samples and multisource evaluations with experts and AI, focusing on history-taking and diagnostic reasoning. Specificities are evident in comparative settings (AI vs humans, multimodel comparisons) and statistical methods such as bootstrap, false discovery rate (FDR), and ICC. Across studies, 5 key evaluation aspects were assessed using objective metrics. Clinical accuracy and knowledge were measured using top-k accuracy [19,21], GTPA@k [25], information coverage [22,43], and span-level *F*_1_-score [18], capturing high diagnostic accuracy and knowledge acquisition. Authenticity was evaluated using hallucination rate [17] and fidelity metrics [18]. Interaction experience was assessed via CUQ [24] and Anthropomorphism Score [17], reflecting communication quality and user perception. Robustness and consistency were quantified using *κ* [30] and ICC [39]. System performance was captured through latency, failure rate, and confusion rate [21,49]. These categories collectively enable a comprehensive assessment of both technical and experiential quality, highlighting the systems’ effectiveness while indicating areas for further standardization and evaluation refinement.

### Finding for RQ4: What Public Datasets Are Available, and What Are Their Characteristics for Training, Simulating, and Evaluating Medical History-Taking in Virtual Patient Systems?

A variety of datasets support the development and evaluation of virtual patient systems, ranging from real-world EHRs to structured synthetic clinical scenarios. Table 4 summarizes key datasets used in recent studies, which are either publicly available or accessible through formal application processes. These datasets provide diverse clinical data resources for effective clinical training and evaluation.

MIMIC-III (Medical Information Mart for Intensive Care-III) is a comprehensive publicly available medical dataset, containing detailed EHR data from over 40,000 intensive care unit (ICU) patients [10]. It covers multiple disease types, including internal medicine and neurological and rheumatological conditions, serving as a critical resource for virtual patient system development. However, its ICU-focused nature, primarily reflecting acute or severe diseases, limits its applicability to noncritical care scenarios, such as outpatient consultations, mental health disorders, or chronic disease management [10].

DDxPlus provides clinical dialogue data for respiratory diseases [25], suitable for training virtual patient systems in specific domains within internal medicine. The iCraft-MD dataset focuses on dermatological cases [25], and RareBench contains region-specific subsets of rare and multiple disease types [25]. While valuable for specialized applications, their scope is limited for building generalized, multitask virtual patient systems.

The medical-NLP corpus provides a broad range of clinical dialogues and records [18], while CCKS 2019 offers a Chinese medical KG dataset, enabling multilingual history-taking simulations [17,56]. The Open-i dataset includes multimodal chest X-ray images and textual descriptions, supporting dynamic image generation alongside history-taking [47]. MedQA provides multiple-choice and long-form medical question-answering data, useful for fine-tuning LLMs for diagnostic dialogues [45]. These datasets enhance multimodal and question-answering capabilities, though their coverage is narrower than MIMIC-III.

In summary, current datasets primarily focus on critical care or specific medical domains, with limited publicly available, diverse resources. These datasets have limitations in generalizability, multitask applicability, and multilingual support for virtual patient systems. Combining MIMIC-III (broad coverage) [10], DDxPlus and iCraft-MD (specialized domains) [25], and CCKS 2019 (multilingual support) [17,56] provides partial coverage. However, further development of diverse, emotionally annotated, and non-English datasets is needed to enhance system generalizability and conversational fidelity.

**Table 4:** Datasets used in virtual patient systems.

Dataset Name	Index	Description	Access requirements
MIMIC-III^a^	[10]	EHR^b^ database from Beth Israel Deaconess Medical Center, over 40,000 ICU^c^ patients (2001-2012), including vital signs, medications, laboratory results, diagnostic codes.	Access via PhysioNet; requires CITI training and DUA [52].
DDxPlus	[25]	Synthetic dataset for respiratory diseases, with clinical dialogues and diagnoses.	CC-BY^d^; publicly available, allows commercial use with attribution [53].
iCraft-MD	[25]	Synthetic dermatology dataset from public medical question banks and expert cases.	MIT License; publicly available [54].
RareBench	[25]	Rare disease dataset with regional subsets (Europe, Canada, and China).	Apache-2.0; publicly available [55].
medical-nlp	[18]	Medical NLP^e^ corpus with clinical dialogues and records.	GNU General Public License v3.0; publicly available on GitHub [18].
CCKS 2019	[17]	Chinese medical knowledge graph dataset with entity recognition, relation extraction, QA^f^ tasks.	Research use only; cite source [56].
Open-i	[47]	Multimodal dataset with 3314 chest x-ray images and textual descriptions.	Open Data Commons Open Database License; publicly available via NIH Open-i project [57].
MedQA	[45]	Multiple-choice and long-form medical QA dataset for diagnostics.	Publicly available via GitHub [45].

^a^MIMIC-III: Medical Information Mart for Intensive Care-III.

^b^EHR: electronic health record.

^c^ICU: intensive care unit.

^d^CC-BY: Creative Commons Attribution.

^e^NLP: natural language processing.

^f^QA: question-answering.

## Discussion

### Principal Findings

Current LLM-based virtual patient systems exhibit significant limitations in disease coverage, complex case simulation, multimorbidity representation, specialty applicability, multimodal capabilities, and standardization of evaluation metrics, indicating a need for systematic optimization to enhance clinical fidelity, educational adaptability, and interaction quality.

### Limitations and Future Directions in Virtual Patient Type Simulation Research

The systematic review reveals several limitations in current virtual patient systems regarding disease coverage, case complexity modeling, and support across medical specialties [20,32]. First, research is heavily concentrated on internal medicine (eg, gastrointestinal, respiratory, and metabolic disorders) and mental health disorders, accounting for over half of the studies [15,32]. This focus reflects the strengths of LLMs in language-driven tasks and the availability of relevant data, but it highlights significant gaps in surgical and orthopedic, ophthalmological, and dermatological domains. Scenarios requiring procedural operations, image recognition, or multimodal interactions are underrepresented, with current systems lacking effective modeling mechanisms [16,43]. This finding aligns with RQ1, indicating that most simulations focus on specific disease categories, leaving certain specialty areas insufficiently covered.

Although some studies involve multiple disease types, current virtual patient systems mainly use single-disease trajectories, with each virtual patient representing only one disease [21,25]. multimorbidity simulations—patients with 2 or more coexisting chronic or acute diseases—are limited. Clinical multimorbidity requires complex decisions, including drug interactions, overlapping symptoms, and conflicting management priorities. Dataset analysis for RQ4 shows most datasets focus on a single primary disease, lacking comprehensive multimorbidity cases. Without simulating this complexity, virtual patient systems have limited use in advanced medical education and clinical reasoning. Future research should emphasize role construction and dialogue design for multimorbidity scenarios to support integrated management across multiple diseases.

Furthermore, current systems exhibit instability in simulating vague or atypical symptoms (eg, chronic fatigue, low mood, attention deficits), demonstrating weak extraction of unstructured chief complaints and incoherent reasoning processes, which limits support for comprehensive clinical assessments [10,31]. Incorporating KGs and CoT reasoning mechanisms may improve knowledge organization and causal chain construction, enhancing models’ reasoning and response capabilities in complex clinical scenarios [58,59]. Open-source KGs, such as UMLS (Unified Medical Language System) [60], SNOMED-CT (Systematized Nomenclature of Medicine - Clinical Terms) [61] can be integrated to provide structured, high-coverage medical knowledge. Leveraging these KGs allows LLMs to reference verified entities and relationships during dialogue generation, reducing hallucinations and enhancing reasoning in multimorbidity or complex symptom scenarios.

Additionally, coverage of specialty domains remains limited, particularly in surgical and orthopedic, ophthalmological, and dermatological contexts requiring procedural or visual recognition skills. These fields show constraints in case complexity, multimodal interaction support, and procedural training, limiting educational adaptability and clinical fidelity [43]. This observation is consistent with findings from RQ1 and RQ4, indicating that certain specialty cases and complex patient scenarios remain underrepresented.

In summary, current virtual patient systems require systematic optimization in disease coverage balance, case complexity, multimorbidity simulation, specialty applicability, multimodal capabilities, and interaction depth. These improvements are expected to enhance clinical fidelity, educational adaptability, and natural human-computer interaction, providing a foundation for advancing intelligent medical education and clinical decision support systems.

### Technical Challenges and Solutions of LLM-Based Virtual Patient Simulation

In virtual patient simulation, prompts guide LLMs to generate dialogues conforming to specific role characteristics. However, overly long prompts lead to information loss, affecting dialogue quality and coherence. Specifically, GPT-style models based on autoregressive decoder architectures process prompts sequentially from left to right, prioritizing information at the beginning (primacy effect) and end (recency effect), while middle information may be ignored or inadequately processed, impacting content completeness and accuracy [62]. Additionally, few-shot learning maintains role settings using limited historical dialogues, but balancing information completeness and avoiding overload in multiturn complex dialogues remains challenging. To address this, core information should be prioritized at the prompt’s beginning and end, with emotional content placed in the middle to ensure accurate and consistent information transmission.

Compared to single-prompt methods, multiagent frameworks decompose dialogue generation into modular components, with agents handling specific functions such as patient history or simulated emotions, or leveraging adversarial training to enhance dialogue quality [18,21,24]. However, multiagent systems increase computational costs, as each agent independently calls APIs, leading to higher financial burdens. Additionally, fine-tuning using older APIs may cause “memory loss,” affecting dialogue context continuity. Dialogue saving and restoration mechanisms are necessary to ensure information consistency. To reduce costs and improve efficiency, dialogue compression and summarization mechanisms can simplify context input, and noncritical tasks can be assigned to lower-cost APIs to balance performance and expense.

Research on fine-tuning GPT-style models and integrating KGs remains limited. Liu et al [17] proposed a SFT strategy using synthetic medical records and manually annotated dialogues, significantly enhancing the realism and anthropomorphism of patient history collection dialogues on the Qwen-72B model (Alibaba Cloud), reducing Hallucination Rate from 3.71% to 0.31%. However, their training process did not explicitly embed disease names into the Transformer model, limiting specific disease knowledge mastery. Prior studies indicate that incorporating disease names as labels or input features improves semantic understanding and generalization, particularly for simulating patient histories for specific diseases [58,59]. Combining CoT methods with fine-tuning to guide step-by-step reasoning for complex medical knowledge is underexplored and presents a potential direction for medical dialogue systems.

KGs systematically represent structured medical knowledge, but there is a lack of studies comparing disease embeddings trained by LLMs with KG embeddings to evaluate differences in knowledge representation and reasoning capabilities. KGs’ clear entity and relation structures complement LLMs’ limitations in sparse knowledge or verification, while LLM-trained embeddings as a baseline reveal constraints in semantic understanding and improvements from KG enhancement. Future research should establish a unified evaluation framework integrating supervised fine-tuning, LoRA, and CoT reasoning to explore the complementary roles of KG and LLM embeddings, advancing medical dialogue models in logical reasoning and knowledge accuracy.

### Evaluation Design Suggestions and Summary of Metrics

Evaluation methods and metrics in LLM-based virtual patient systems are diverse, reflecting the multidimensional nature of performance and educational outcomes. However, the lack of standardized frameworks hinders cross-study comparison and generalizability. Diagnostic accuracy is often measured by top-k accuracy (eg, 65% [19]) and GTPA@k [25], though these coarse metrics may not capture system capabilities. Information Coverage and Hallucination Rate assess retrieval fidelity but usually rely on manual verification. Interaction quality metrics—such as semantic similarity (0.7589 [18]) and user scores (eg, CUQ=77 [22])—highlight interactivity but are limited by subjectivity and small samples. External studies also report high usability, such as SUS=88.1[31], a 10-item scale measuring ease of use, confidence, and learnability, reinforcing the systems’ educational value despite inconsistent evaluation standards.

Moreover, system performance assessment remains insufficient. Some studies report response delays affecting conversation naturalness, but lack quantitative measures or systematic evaluations. The absence of standardized key performance indicators exacerbates framework fragmentation, hindering effective implementation and broader application in medical education and effective clinical training [41].

To address fragmented metrics, a unified and scientifically grounded evaluation framework is necessary. Key performance indicators with recommended thresholds are proposed to guide system design and assessment:

- Top-1 accuracy (≥0.80): AMIE achieved a top-1 accuracy of 0.65, outperforming primary care physicians (0.53) [19], while *GTPA*@1 reached 0.96 [25]. A threshold of 0.80 ensures reliable diagnostic performance.
- Hallucination rate (≤0.05): GPT-4o demonstrated a Hallucination Rate of 0.31% [17], with rates below 5% reported in [19], supporting medical safety standards.
- Information coverage (≥0.50): coverage of critical history items was 33.89% [43], indicating room for improvement. A 50% threshold ensures adequate information capture.
- Empathy and Anthropomorphism Score (≥0.75, standardized 0-1 scale): GPT-4o scored 0.87 [17], and [37] reported approximately 0.76, indicating desired human-like interaction and empathy.
- Usability (SUS≥80, CUQ≥75): SUS of 88.1 [59] and CUQ of 77 [22] meet standards for satisfactory usability.
- Robustness (leak resistance≥0.90): a value of 0.9412 was reported in [18], indicating compliance with privacy and ethical requirements.
- Rater consistency (*κ* and ICC≥0.80): high interrater reliability was observed, with ICC=0.924 [39] and *κ*=0.832 [8].

### Challenges of Data Diversity: Limitations in Corpus Coverage and Adaptability

Existing datasets for training and evaluating LLM-based virtual patient systems are diverse, encompassing real-world EHRs, synthetic clinical scenarios, and multimodal or multilingual resources [10,17,18,25,45,47,56]. Table 4 summarizes key datasets, which are either publicly available or accessible through formal application processes. Analysis reveals several limitations impacting system development.

Mainstream datasets like MIMIC-III primarily reflect intensive care scenarios, containing records from ICU patients with acute or critical conditions, often within internal medicine or neurological and rheumatological categories [10]. This bias limits generalizability to nonacute settings, such as outpatient consultations, mental health disorders, or chronic disease management. Specialized datasets, including DDxPlus (internal medicine), iCraft-MD (dermatological), and RareBench (rare and multiple disease types), provide tailored resources [25]. While valuable for specific domains, their narrow coverage limits suitability for general-purpose or multitask system training.

Linguistic and cultural diversity is limited, as most corpora are English-based and originate from Western health care systems [17,18,45,47,56]. The lack of datasets in other languages, such as Chinese [17,56], and integration with local KGs or region-specific disease contexts constrains performance in multilingual and cross-cultural environments. Modality limitations are evident; most datasets provide textual information and lack multimodal inputs like images, speech, or physiological signals [47], restricting interaction realism and diagnostic reasoning.

Data accessibility and format heterogeneity affect usability. Access requirements, annotation styles, field definitions, and dialogue formats vary [10,17,18,25,45,47,56], hindering integration and comprehensive training. Standardization of data formats and unified interfaces is necessary to reduce development costs and support broader adoption. Additionally, task alignment poses challenges, as datasets like MedQA [45] are structured for multiple-choice or question-answering tasks, requiring extensive adaptation for dialogue generation.

Combining broadly covering datasets like MIMIC-III [10] with domain-specific (DDxPlus, iCraft-MD, RareBench) [25] and multilingual or multimodal datasets (CCKS 2019 [17,56], Open-i [47], medical-nlp [18], MedQA [45]) can partially address gaps. However, developing diverse, emotionally annotated, non-English, and multimodal datasets is essential to enhance generalizability, robustness, and interaction fidelity.

### Conclusion

This systematic review, conducted per PRISMA 2020 guidelines, evaluated studies (January 2020-August 18, 2025) on LLM-based virtual patient systems for medical history collection, sourced from 9 databases PubMed, Scopus, Web of Science, IEEE Xplore, ACM Digital Library, Springer, ERIC, arXiv, and ACL Anthology. Following rigorous screening, deduplication, and quality appraisal, 6 high-quality and 33 moderate-quality studies were included, addressing 4 research questions, simulated patient types, performance-enhancing technologies, experimental designs, and evaluation metrics.

Key findings include (1) systems primarily simulate internal medicine and mental health disorders (acute and chronic), with limited coverage of rare and multiple disease types, multimorbidity, and specialties like surgical and orthopedic, neurological and rheumatological, dermatological, and ophthalmological, restricting applicability in complex clinical reasoning and education [15,16,32,43]. (2) Technologies such as role-based prompts, few-shot learning, multiagent frameworks, KGs, and fine-tuning (eg, SFT, LoRA, CoT, RLAIF+MoM) enhance dialogue coherence, retrieval accuracy (+16.02% with KGs) [10], and high diagnostic accuracy, while multimodal integration (eg, speech) improves immersion [18,25]. (3) Evaluations involved medical students and practitioners, using mixed methods (top-k accuracy, *F*_1_-score, SUS, CUQ, and expert ratings) with comparisons across AI models, physicians, or prompt variations; small sample sizes (10-50 students and 3-10 experts) and inconsistent metrics limit generalizability [19,40]. (4) Systems demonstrated high diagnostic accuracy: top-k accuracy 0.45-0.98, information coverage 33.89%-94.4%, Hallucination Rate 0.31%-5%, and high usability (SUS≥80), often outperforming junior physicians [18,43]. Dataset limitations (eg, MIMIC-III ICU bias, restricted access, low multilingual and multimodal diversity) hinder cross-study comparability [10,17,25].

The key discussion points are summarized: (1) Disease coverage is imbalanced, favoring internal medicine and mental health disorders over surgical and orthopedic, dermatological, ophthalmological, and multimorbidity scenarios, limiting effective clinical training [16,43]. Future systems should prioritize multimorbidity and diverse patient populations (cultural and linguistic) to enhance realism [17,56]. (2) Prompt design endures information loss in long prompts; placing critical information at prompt ends and using dialogue compression or multiagent frameworks can mitigate this [18,60]. KG-LLM integration and fine-tuning improve performance, with potential for further gains via hybrid KG-CoT approaches [58,59]. (3) Fragmented evaluation frameworks, inconsistent metrics, and small participant pools reduce reliability. A standardized framework with thresholds (eg, top-1 accuracy≥0.80, hallucination rate≤0.05, SUS≥80, CUQ≥75,κ and ICC≥0.80) and larger samples (50-100 students, 5-10 experts) is needed [19,39]. (4) Dataset biases (eg, ICU focus), format heterogeneity, and privacy restrictions limit inclusivity. Open-access, ethically compliant, multimodal, and multilingual datasets are essential for equitable systems [10,45,47].

Future research should focus on large-scale longitudinal studies, standardized evaluation metrics, diverse open-access datasets (eg, UMLS [60] and SNOMED-CT [61]), and advanced integration of KGs, multimodal training, and optimized prompts to enhance the realism, high diagnostic accuracy, and fairness of LLM-based virtual patient systems in medical education [17,18,59].

## Appendix

Multimedia Appendix 1 Database Search Strategies.

Multimedia Appendix 2 PRISMA Checklist.

Multimedia Appendix 3 Joanna-Briggs Institute Quality Assessment Questionnaire.

Multimedia Appendix 4 Multidimensional assessment table.

Multimedia Appendix 5 Quality Assessment Result.

Multimedia Appendix 6 Risk of Bias Assessment for 39 Included Studies.

Multimedia Appendix 7 Calculation Formulas for Evaluation Metrics.

Multimedia Appendix 8 Other file screening process, source files, etc.

## Abbreviations

AI: artificial intelligence
API: application programming interface
AS: Anthropomorphism Score
BERT: Bidirectional Encoder Representations from Transformers
CoT: chain-of-thought
CUQ: Chatbot Usability Questionnaire
EHR: electronic health record
FDR: false discovery rate
ICC: intraclass correlation coefficient
ICU: intensive care unit
KG: knowledge graph
LLM: large language model
LLMDP: LLM-based digital patient system
LoRA: Low-Rank Adaptation
MIMIC-III: Medical Information Mart for Intensive Care-III
OSCE: Objective Structured Clinical Examination
PCP: primary care physician
PRISMA: Preferred Reporting Items for Systematic Reviews and Meta-Analyses
RAG: retrieval-augmented generation
RCT: randomized controlled trial
RLHF: reinforcement learning with human feedback
RQ: research question
SFT: supervised fine-tuning
SNOMED-CT: Systematized Nomenclature of Medicine - Clinical Terms
SUS: System Usability Scale
UMLS: Unified Medical Language System
