# Supplementary material for: Large Language Model–Based Virtual Patient Systems for History-Taking in Medical Education: Comprehensive Systematic Review
Source: JMIR Med Inform. 2026 Jan 2;14:e79039. doi: 10.2196/79039 (PMC12811743; doi:10.2196/79039)
Supplement: Multimedia Appendix 1 [file medinform_v14i1e79039_app1.docx]

## Appendix 1.Database Search Strategies

Complete and Reproducible Search Strategies for Academic Databases

| **Database** | **Search Query and Details** |
| --- | --- |
|  |  |
| PubMed | **Query**: ("virtual patient"[All Fields] OR "simulated patient"[All Fields] OR "AI patient"[All Fields] OR "conversational patient"[All Fields] OR "chatbot patient"[All Fields] OR "intelligent virtual agent"[All Fields] OR "dialogue agent"[All Fields]) AND ("large language model"[All Fields] OR "LLM"[All Fields] OR "ChatGPT"[All Fields] OR "GPT-4"[All Fields] OR "GPT"[All Fields] OR "transformer model"[All Fields] OR "generative AI"[All Fields] OR "AI-powered tutor"[All Fields] OR "natural language generation"[All Fields])  **Date Range**: 2020-01-01 to 2025-08-18  **Document Type**} Article, Review, Clinical Study, Case Reports  **Language**: English  **Remarks**: Humans, Q1–Q4 journals |
| Scopus | **Query**: ("virtual patient" OR "simulated patient" OR "AI patient" OR "conversational patient" OR "chatbot patient" OR "intelligent virtual agent" OR "dialogue agent") AND ("large language model" OR "LLM" OR "ChatGPT" OR "GPT-4" OR "GPT" OR "transformer model" OR "generative AI" OR "AI-powered tutor" OR "natural language generation")  **Date Range**: 2020-01-01 to 2025-08-18  **Document Type**: Article, Conference Paper  **Language**: English  **Remarks**: All Fields |
| Web of Science | **Query**: ("virtual patient" OR "simulated patient" OR "AI patient" OR "conversational patient" OR "chatbot patient" OR "intelligent virtual agent" OR "dialogue agent") AND ("large language model" OR "LLM" OR "ChatGPT" OR "GPT-4" OR "GPT" OR "transformer model" OR "generative AI" OR "AI-powered tutor" OR "natural language generation")  **Date Range**: 2020-01-01 to 2025-08-18  **Document** Type: Meeting, Article, Early Access  **Language**: English  **Remarks**: Exclude Preprint Citation Index |
| IEEE Xplore | **Query**: ("virtual patient" OR "simulated patient" OR "AI patient" OR "conversational patient" OR "chatbot patient" OR "intelligent virtual agent" OR "dialogue agent") AND ("large language model" OR "LLM" OR "ChatGPT" OR "GPT-4" OR "GPT" OR "transformer model" OR "generative AI" OR "AI-powered tutor" OR "natural language generation")  **Date Range**: 2020-01-01 to 2025-08-18  **Document Type**: Journal Article, Conference Paper  **Language**: English  **Remarks**: – |
| ACM | **Query**: ("virtual patient" OR "simulated patient" OR "AI patient" OR "conversational patient" OR "chatbot patient" OR "intelligent virtual agent" OR "dialogue agent") AND ("large language model" OR "LLM" OR "ChatGPT" OR "GPT-4" OR "GPT" OR "transformer model" OR "generative AI" OR "AI-powered tutor" OR "natural language generation")  **Date Range**}: 2020-01-01 to 2025-08-18  **Document Type**}: Research Article  **Language**}: English  **Remarks**}: – |
| Springer | **Query**: ("virtual patient" OR "simulated patient" OR "AI patient" OR "conversational patient" OR "chatbot patient" OR "intelligent virtual agent" OR "dialogue agent")  **Date Range**: 2020-01-01 to 2025-08-18  **Document Type**: Article, Research Article, Conference Paper  **Language**: English  **Remarks**: Content type limited |
| ERIC | **Query**: ("virtual patient" OR "simulated patient" OR "AI patient" OR "conversational patient" OR "chatbot patient" OR "intelligent virtual agent" OR "dialogue agent")  **Date Range**: 2016-01-01 to 2025-08-18  **Document Type**: Journal Article, Report  **Language**: English  **Remarks**: 2016–2019 removed |
| arXiv | **Query**: ("virtual patient" OR "simulated patient" OR "AI patient" OR "conversational patient" OR "chatbot patient" OR "intelligent virtual agent" OR "dialogue agent")  **Date Range**: 2020-01-01 to 2025-08-18  **Document** **Type**: Preprint  **Language**: English  **Remarks**: Include cross-listed papers |
| ACL Anthology | **Query**: ("virtual patient" OR "simulated patient" OR "AI patient" OR "conversational patient" OR "chatbot patient" OR "intelligent virtual agent" OR "dialogue agent")  **Date Range**: 2020-01-01 to 2025-08-18  **Document** **Type**: Conference Paper, Workshop Paper  **Language**: English  **Remarks**: – |
