# Supplementary material for: Large Language Model–Based Virtual Patient Systems for History-Taking in Medical Education: Comprehensive Systematic Review
Source: JMIR Med Inform. 2026 Jan 2;14:e79039. doi: 10.2196/79039 (PMC12811743; doi:10.2196/79039)
Supplement: Multimedia Appendix 3 [file medinform_v14i1e79039_app3.docx]

## Appendix 2. JBI Quality Assessment Questionnaire

JBI Quality Assessment Questionnaire with Scoring Criteria

| **Question** | **0** | **1** | **2** |
| --- | --- | --- | --- |
|  |  |  |  |
| **Q1: Clarity of Methods**  Are the study methods described in sufficient detail to allow replication? | □Poor | □Moderate | □Excellent |
| **Q2: Dataset Transparency**  Is the dataset used in the study clearly described and accessible? | □Poor | □Moderate | □Excellent |
| **Q3: Completeness of Evaluation**  Are the system evaluation methods comprehensive and appropriate? | □Poor | □Moderate | □Excellent |
| **Q4: Innovation / Integration**  Does the study present innovative methods or integrate multiple modalities? | □Poor | □Moderate | □Excellent |
| **Q5: Reproducibility / Openness**  Are the code, models, or data publicly available to allow replication? | □Poor | □Moderate | □Excellent |
| **Q6: Control or Baseline Comparison**  Does the study include comparisons with baseline methods or control groups? | □Poor | □Moderate | □Excellent |
| **Total Score / Range** | ______ / 12 | | |
| **Overall Quality** | Low / Moderate / High | | |

***Note*:** 0 = Poor (low quality / insufficient information), 1 = Moderate (adequate quality / some information), 2 = Excellent (high quality / fully reported)
