# Supplementary material for: Large Language Model–Based Virtual Patient Systems for History-Taking in Medical Education: Comprehensive Systematic Review
Source: JMIR Med Inform. 2026 Jan 2;14:e79039. doi: 10.2196/79039 (PMC12811743; doi:10.2196/79039)
Supplement: Multimedia Appendix 4 [file medinform_v14i1e79039_app4.docx]

## Appendix 3. Multi-dimensional Assessment Table

Multi-dimensional Assessment Table for LLM-based Virtual Patient Studies

| **Dimension** | **Question** | **Yes** | **No** | **Unclear** | **N/A** |
| --- | --- | --- | --- | --- | --- |
|  |  |  |  |  |  |
| Focus on LLM Virtual Patient | 1.1 Does the study describe the disease type simulated (e.g., internal, surgery)? | □ | □ | □ | □ |
|  | 1.2 Does the study include multiple diseases or complex cases? | □ | □ | □ | □ |
|  | 1.3 Does the study cover different disease stages? | □ | □ | □ | □ |
| Model Specification | 2.1 Is the LLM model type specified (GPT, LLaMA)? | □ | □ | □ | □ |
|  | 2.2 Is the training or fine-tuning method described (Few-shot, Fine-tune)? | □ | □ | □ | □ |
|  | 2.3 Does it include prompt engineering or role-playing? | □ | □ | □ | □ |
|  | 2.4 Is RLHF or domain-specific fine-tuning applied? | □ | □ | □ | □ |
|  | 2.5 Is multimodal integration used (text, audio, image)? | □ | □ | □ | □ |
|  | 2.6 Is system architecture described (frontend, backend, API)? | □ | □ | □ | □ |
|  | 2.7 Are personalization or adaptive mechanisms included? | □ | □ | □ | □ |
|  | 2.8 Are quality controls applied (accuracy, ethics)? | □ | □ | □ | □ |
| Evaluation | 3.1 Is educational effect assessed (H&P skills, clinical decision)? | □ | □ | □ | □ |
|  | 3.2 Is user experience or technical performance assessed? | □ | □ | □ | □ |
|  | 3.3 Are comparative studies conducted (vs real SP)? | □ | □ | □ | □ |
| Dataset | 4.1 Is a specific dataset type used (text, image)? | □ | □ | □ | □ |
|  | 4.2 Is dataset size adequate or large (≥ 100 samples)? | □ | □ | □ | □ |
|  | 4.3 Is the data source specified (public DB, synthetic)? | □ | □ | □ | □ |
|  | 4.4 Is data labeling or quality control described? | □ | □ | □ | □ |
|  | 4.5 Is the data used for teaching, validation, or research? | □ | □ | □ | □ |
| **Overall** | Include / Exclude / Seek for more information | □ | □ | □ | □ |
| **Comments:** |  | | | | |
