# Supplementary material for: Large Language Model–Based Virtual Patient Systems for History-Taking in Medical Education: Comprehensive Systematic Review
Source: JMIR Med Inform. 2026 Jan 2;14:e79039. doi: 10.2196/79039 (PMC12811743; doi:10.2196/79039)
Supplement: Multimedia Appendix 5 [file medinform_v14i1e79039_app5.docx]

## Appendix 6. Quality Assessment Result

| **Ref No** | **Q1** | **Q2** | **Q3** | **Q4** | **Q5** | **Q6** | **Score** | **Ranking** |
| --- | --- | --- | --- | --- | --- | --- | --- | --- |
|  | | | | | |  |  |  |
| [4] | 2 | 0 | 1 | 2 | 1 | 2 | 8 | Medium |
| [29] | 2 | 1 | 2 | 2 | 1 | 0 | 8 | Medium |
| [14] | 2 | 1 | 1 | 2 | 1 | 0 | 7 | Medium |
| [22] | 2 | 1 | 2 | 2 | 1 | 0 | 8 | Medium |
| [21] | 2 | 1 | 1 | 2 | 2 | 0 | 8 | Medium |
| [51] | 2 | 0 | 1 | 2 | 0 | 0 | 5 | Medium |
| [2] | 2 | 0 | 1 | 2 | 0 | 1 | 6 | Medium |
| [40] | 2 | 2 | 1 | 2 | 2 | 0 | 9 | High |
| [23] | 2 | 1 | 2 | 2 | 1 | 0 | 8 | Medium |
| [39] | 2 | 0 | 1 | 2 | 0 | 0 | 5 | Medium |
| [41] | 2 | 0 | 1 | 2 | 0 | 0 | 5 | Medium |
| [37] | 2 | 0 | 1 | 2 | 1 | 0 | 6 | Medium |
| [43] | 2 | 0 | 1 | 2 | 0 | 0 | 5 | Medium |
| [1] | 2 | 1 | 2 | 1 | 1 | 0 | 7 | Medium |
| [52] | 2 | 0 | 1 | 2 | 0 | 0 | 5 | Medium |
| [47] | 2 | 0 | 1 | 2 | 0 | 1 | 6 | Medium |
| [25] | 2 | 1 | 1 | 2 | 0 | 1 | 7 | Medium |
| [30] | 2 | 1 | 2 | 2 | 1 | 2 | 10 | High |
| [32] | 2 | 1 | 1 | 1 | 0 | 0 | 5 | Medium |
| [42] | 2 | 1 | 1 | 2 | 1 | 0 | 7 | Medium |
| [50] | 2 | 1 | 2 | 2 | 1 | 0 | 8 | Medium |
| [28] | 2 | 0 | 1 | 2 | 1 | 0 | 6 | Medium |
| [48] | 2 | 0 | 1 | 2 | 1 | 2 | 8 | Medium |
| [49] | 2 | 0 | 1 | 2 | 1 | 0 | 6 | Medium |
| [36] | 2 | 1 | 1 | 2 | 0 | 0 | 6 | Medium |
| [24] | 0 | 0 | 0 | 0 | 0 | 0 | 10 | High |
| [33] | 2 | 1 | 1 | 2 | 1 | 0 | 7 | Medium |
| [6] | 2 | 1 | 2 | 2 | 1 | 1 | 9 | High |
| [31] | 2 | 1 | 2 | 2 | 1 | 1 | 9 | High |
| [38] | 2 | 0 | 1 | 2 | 0 | 0 | 5 | Medium |
| [34] | 2 | 1 | 1 | 2 | 1 | 1 | 8 | Medium |
| [44] | 2 | 1 | 2 | 2 | 1 | 2 | 10 | High |
| [45] | 2 | 1 | 1 | 2 | 1 | 0 | 7 | Medium |
| [35] | 2 | 0 | 1 | 2 | 0 | 0 | 5 | Medium |
| [26] | 2 | 0 | 1 | 2 | 0 | 0 | 5 | Medium |
| [3] | 2 | 1 | 1 | 2 | 1 | 0 | 7 | Medium |
| [27] | 2 | 0 | 1 | 2 | 0 | 1 | 6 | Medium |
| [46] | 2 | 0 | 1 | 2 | 0 | 1 | 6 | Medium |
| [5] | 2 | 1 | 1 | 2 | 0 | 0 | 6 | Medium |

***Note*:** Q1=Method Clarity; Q2=Dataset Transparency; Q3=System Evaluation Completeness; Q4=Innovation or Integration Degree; Q5=Reproducibility and Openness; Q6=Comparative Study or Baseline Comparison；High (9–12 points), Medium (5–8 points), and Low (0–4 points).
