# Supplementary material for: Large Language Model–Based Virtual Patient Systems for History-Taking in Medical Education: Comprehensive Systematic Review
Source: JMIR Med Inform. 2026 Jan 2;14:e79039. doi: 10.2196/79039 (PMC12811743; doi:10.2196/79039)
Supplement: Multimedia Appendix 7 [file medinform_v14i1e79039_app7.docx]

## Appendix 7. Risk of Bias Assessment for 39 Included Studies

| **Ref No** | **Selection and Re- porting Bias** | **Implementation Bias** | **Evaluation Bias** | **Data Bias Reporting** | **Com- pleteness Bias** |
| --- | --- | --- | --- | --- | --- |
|  | | | | | |
| [18] | Low | Moderate | Low | Low | High |
| [28] | High | Moderate | Moderate | High | Moderate |
| [8] | Moderate | Moderate | Low | Moderate | Moderate |
| [21] | Moderate | Moderate | Low | Low | Moderate |
| [20] | Moderate | Moderate | Low | Low | Moderate |
| [50] | Moderate | Moderate | Moderate | High | Moderate |
| [16] | Low | Moderate | Moderate | Moderate | Moderate |
| [39] | Moderate | Moderate | Moderate | High | Moderate |
| [22] | Moderate | Moderate | Moderate | Low | Moderate |
| [38] | Moderate | Moderate | Moderate | Low | Moderate |
| [40] | High | Moderate | Moderate | High | High |
| [36] | Moderate | Moderate | Moderate | Moderate | High |
| [42] | Moderate | Low | Low | Low | Low |
| [15] | Moderate | Moderate | Moderate | Low | Moderate |
| [51] | Moderate | Moderate | Moderate | High | High |
| [46] | Moderate | Moderate | Moderate | Low | High |
| [24] | Moderate | Moderate | Moderate | Moderate | Low |
| [29] | Moderate | Moderate | Moderate | Low | Moderate |
| [31] | Moderate | Low | Moderate | Low | Low |
| [41] | Moderate | Moderate | Low | High | High |
| [49] | Moderate | Moderate | Moderate | Low | Moderate |
| [27] | Moderate | Moderate | Moderate | High | High |
| [47] | Moderate | Low | Moderate | Low | Moderate |
| [48] | Moderate | Moderate | Low | Low | Moderate |
| [35] | Low | Moderate | Moderate | High | Moderate |
| [23] | Moderate | Moderate | Low | Low | Moderate |
| [32] | Moderate | Moderate | Low | Low | Moderate |
| [10] | Moderate | Low | Low | Low | Low |
| [30] | Moderate | Moderate | Low | Low | Moderate |
| [37] | High | Moderate | Moderate | High | Low |
| [33] | Moderate | Moderate | Moderate | High | High |
| [43] | Low | Moderate | Low | Low | Moderate |
| [44] | Moderate | Moderate | Moderate | Moderate | Moderate |
| [34] | Moderate | Low | Low | Low | Low |
| [25] | Moderate | Low | Low | Low | Low |
| [17] | Moderate | Moderate | Low | Low | Moderate |
| [26] | Moderate | Moderate | Low | Low | Moderate |
| [5] | Moderate | Moderate | Low | Low | Moderate |
| [19] | Low | Low | Low | Low | Low |
