# Supplementary material for: Large Language Model–Based Virtual Patient Systems for History-Taking in Medical Education: Comprehensive Systematic Review
Source: JMIR Med Inform. 2026 Jan 2;14:e79039. doi: 10.2196/79039 (PMC12811743; doi:10.2196/79039)
Supplement: Multimedia Appendix 8 [file medinform_v14i1e79039_app8.zip › Appendix 5.docx]

## Appendix 5. Calculation Formulas for Evaluation Metrics

Formulas for Key Evaluation Metrics in Virtual Patient Systems

| **Metric** | **Formula** | **Description** | **Reference** |
| --- | --- | --- | --- |
|  | | | |
| **Clinical Accuracy and Knowledge** | | | |
| Top-k Accuracy | $\frac{\# cases where correct diagnosis in top k}{Total cases}$ | Proportion of cases where the correct diagnosis is among the top k predictions, e.g., 81.8% Top-1 for August AI. | [5,46] |
| GTPA@k | $\frac{\# cases where ground truth pathology in top k}{Total cases}$ | Accuracy of pathology predictions in top k, e.g., 0.96 GTPA@1 for GPT-4o on DDxPlus. | [26] |
| Span-level F1 Score | $F1=2\cdot\frac{Precision\cdot Recall}{Precision+Recall}$ | Span-level text matching (knowledge  extraction/evidence retrieval) F1; case: 0.79. | [6] |
| Information Coverage | $\frac{\# relevant info items collected}{Total relevant items}$ | Key history/symptom acquisition rate (e.g., 33.89%). | [44] |
| Hallucination Rate | $\frac{\# responses with false information}{Total responses}$ | Proportion of fictitious/inconsistent  content (e.g., 0.31%; also < 5%). | [3],[5] |
| Relevance/Fidelity | $Cosine Similarity =\frac{\vec{A}\cdot\vec{B}}{\left\vert\vec{A} \right\vert\cdot\left\vert\vec{B} \right\vert}$ | Semantic similarity for relevance/fidelity (e.g., 0.7589/0.8786) | [4] |
| **Communication and Interaction Quality** | | | |
| Flesch Reading Ease | $206.835-1.015\cdot\left( \frac{\mathrm{words}}{\mathrm{sentences}} \right)-84.6\cdot\left( \frac{\mathrm{syllables}}{\mathrm{words}} \right)$ | Readability (higher score indicates easier  reading); used for dialogue clarity. | [6] |
| Flesch-Kincaid Grade | $0.39\cdot\left( \frac{\mathrm{words}}{\mathrm{sentences}} \right)+11.8\cdot\left( \frac{\mathrm{syllables}}{\mathrm{words}} \right)-15.59$ | U.S. grade level for text comprehension, used for response clarity. | [6] |
| CUQ Score | Sum of 16 questions (0–100) | Chatbot usability score, e.g., 77/100 indicates good usability. | [23] |
| SUS Score | $\left( \sum q_{odd}-1+\sum5-q_{even} \right)\cdot2.5$ | System usability | [60] |
| Anthropomorphism | Standardized score (0–1 or 0–5) | Degree of human-like/natural interaction (e.g., 0.87). | [3], [38] |
| **Robustness and Stability** | | | |
| Robustness Score | $1-\frac{\# failures (e.g., leaks)}{Total test cases}$ | Resistance to premature sensitive  information disclosure (e.g., 0.9412). | [4] |
| Cohen’s κ | $\kappa=\frac{p_{0}-p_{e}}{1-p_{e}}$ | Rater consistency/AI-human agreement (e.g., 0.832). | [14] |
| ICC(Reliability) | Intraclass correlation coefficient | Muti-rater consistency ( e.g., ICC=0.924). | [40] |
| ANOVA | $F=\frac{Between-group variance}{Within-group variance}$ | Tests stability across rewrites/scenarios;  high p indicates no significant difference. | [6] |
| **System Performance** | | | |
| Confusion/Clarification  Rate | $\frac{\# \mathrm{clarifications}}{\# turns}$ | Frequency of system clarifications; lower  indicates smoother interaction. | [50] |
| Model Failure Rate | $\frac{\# failed dialoguess}{\# dialoguess}$ | Proportion of interrupted/unprogressable  dialogues; inverse robustness indicator. | [50] |
