# Supplementary material for: Large Language Model–Based Virtual Patient Systems for History-Taking in Medical Education: Comprehensive Systematic Review
Source: JMIR Med Inform. 2026 Jan 2;14:e79039. doi: 10.2196/79039 (PMC12811743; doi:10.2196/79039)
Supplement: Multimedia Appendix 8 [file medinform_v14i1e79039_app8.zip › checklist.pdf]

Appendix 4. PRISMA Checklist

PRISMA 2020 Checklist for Systematic Reviews of LLM-Based Virtual Patient Systems

Topic	No.	Item	Location where item is reported
**TITLE**			
**Title**	1	Identify the report as a systematic review.	Page 1
**ABSTRACT**			
**Abstract**	2	See the PRISMA 2020 for Abstracts checklist	
**INTRODUCTION**			
**Rationale**	3	Describe the rationale for the review in the context of existing knowledge.	Section Introduction
**Objectives**	4	Provide an explicit statement of the objective(s) or question(s) the review addresses.	Section Introduction: RQ1: What types...
**METHODS**			
**Eligibility criteria**	5	Specify the inclusion and exclusion criteria for the review and how studies were grouped for the syntheses.	Section Eligibility Criteria: Population: Population: The population includes...
**Information sources**	6	Specify all databases, registers, websites, organisations, reference lists and other sources searched or consulted to identify studies. Specify the date when each source was last searched or consulted.	Section Information Sources: A systematic literature search was...
**Search strategy**	7	Present the full search strategies for all databases, registers and websites, including any filters and limits used.	Section Search Strategy: Two search strategies were employed...
**Selection process**	8	Specify the methods used to decide whether a study met the inclusion criteria of the review, including how many reviewers screened each record and each report retrieved, whether they worked independently, and if applicable, details of automation tools used in the process.	Section Selection Process and Data Collection: A reviewer with a computer science...

Topic	No.	Item	Location where item is reported
**Data collection process**	9	Specify the methods used to collect data from reports, including how many reviewers collected data from each report, whether they worked independently, any processes for obtaining or confirming data from study investigators, and if applicable, details of automation tools used in the process.	Section Selection Process and Data Collection: The literature screening and data collection process...
**Data items**	10a	List and define all outcomes for which data were sought. Specify whether all results that were compatible with each outcome domain in each study were sought (e.g. for all measures, time points, analyses), and if not, the methods used to decide which results to collect.	No quantitative outcome data were extracted, as this review did not aim to synthesize effect sizes. Instead, the review focused on the methodological characteristics and thematic patterns across studies.
	10b	List and define all other variables for which data were sought (e.g. participant and intervention characteristics, funding sources). Describe any assumptions made about any missing or unclear information.	No quantitative outcome data were extracted, as this review did not aim to synthesize effect sizes. Instead, the review focused on the methodological characteristics and thematic patterns across studies.
**Study risk of bias assessment**	11	Specify the methods used to assess risk of bias in the included studies, including details of the tool(s) used, how many reviewers assessed each study and whether they worked independently, and if applicable, details of automation tools used in the process.	Section Quality and Risk of Bias Assessment: Most studies achieved the highest rating (2 points) ...
**Effect measures**	12	Specify for each outcome the effect measure(s) (e.g. risk ratio, mean difference) used in the synthesis or presentation of results.	No quantitative synthesis or effect measure was applied as this review focused on narrative synthesis.
**Synthesis methods**	13a	Describe the processes used to decide which studies were eligible for each synthesis (e.g. tabulating the study intervention characteristics and comparing against the planned groups for each synthesis (item 5)).	This study is a narrative systematic review and no statistical synthesis was conducted. All studies that met the inclusion criteria were included and used for descriptive analysis and comparison of the results, without further screening for quantitative synthesis.

Topic	No.	Item	Location where item is reported
	13b	Describe any methods required to prepare the data for presentation or synthesis, such as handling of missing summary statistics, or data conversions.	This study is a narrative systematic review and no statistical synthesis analysis was conducted. All studies that met the inclusion criteria were used for the descriptive summary of the results, and no further screening for quantitative synthesis was performed.
	13c	Describe any methods used to tabulate or visually display results of individual studies and syntheses.	To present the similarities and differences among various studies, a structured table was used to organize the key characteristics and results of different studies. Since this study did not conduct quantitative synthesis, there was no 3 Topic No. Item Location where item is reported need to convert the original data or handle missing values.
	13d	Describe any methods used to synthesize results and provide a rationale for the choice(s). If meta-analysis was performed, describe the model(s), method(s) to identify the presence and extent of statistical heterogeneity, and software package(s) used.	None
	13e	Describe any methods used to explore possible causes of heterogeneity among study results (e.g. subgroup analysis, meta-regression).	None
	13f	Describe any sensitivity analyses conducted to assess robustness of the synthesized results.	None
**Reporting bias assessment**	14	Describe any methods used to assess risk of bias due to missing results in a synthesis (arising from reporting biases).	Section Quality and Risk of Bias Assessment: Most studies achieved the highest rating (2 points) ...

Topic	No.	Item	Location where item is reported
**Certainty assessment**	15	Describe any methods used to assess certainty (or confidence) in the body of evidence for an outcome.	Section Quality and Risk of Bias Assessment，Most studies achieved the highest rating ... The assessment determined that it was possible to achieve the goal by optimizing the JBI assessment form and the multi-dimensional assessment form.
**RESULTS**			
**Study selection**	16a	Describe the results of the search and selection process, from the number of records identified in the search to the number of studies included in the review, ideally using a flow diagram.	Selection Study selection results
	16b	Cite studies that might appear to meet the inclusion criteria, but which were excluded, and explain why they were excluded.	Selection Study selection results
**Study characteristics**	17	Cite each included study and present its characteristics.	See the Multimedia Appendix
**Risk of bias in studies**	18	Present assessments of risk of bias for each included study.	Section Quality and Risk of Bias Assessment : Most studies achieved the highest rating ...,And see the Multimedia Appendix
**Results of individual studies**	19	For all outcomes, present, for each study: (a) summary statistics for each group (where appropriate) and (b) an effect estimate and its precision (e.g. confidence/credible interval), ideally using structured tables or plots.	Mainly system literature description: Regarding the four research questions, this part is not applicable. Regarding the evaluation results, due to the significant differences in the research methods employed by the current researchers, they are not applicable.
**Results of syntheses**	20a	For each synthesis, briefly summarise the characteristics and risk of bias among contributing studies.	Selection Study selection results

Topic	No.	Item	Location where item is reported
	20b	Present results of all statistical syntheses conducted. If meta-analysis was done, present for each the summary estimate and its precision (e.g. confidence/credible interval) and measures of statistical heterogeneity. If comparing groups, describe the direction of the effect.	Mainly system literature description: Regarding the four research questions, this part is not applicable. Regarding the evaluation results, due to the significant differences in the research methods employed by the current researchers, they are not applicable.
	20c	Present results of all investigations of possible causes of heterogeneity among study results.	NONE
	20d	Present results of all sensitivity analyses conducted to assess the robustness of the synthesized results.	NONE
**Reporting biases**	21	Present assessments of risk of bias due to missing results (arising from reporting biases) for each synthesis assessed.	Mainly system literature description, this part is not applicable.
**Certainty of evidence**	22	Present assessments of certainty (or confidence) in the body of evidence for each outcome assessed.	Mainly system literature description, this part is not applicable.
**DISCUSSION**			
**Discussion**	23a	Provide a general interpretation of the results in the context of other evidence.	Discussion on challenges and gaps
	23b	Discuss any limitations of the evidence included in the review.	Discussion on challenges and gaps
	23c	Discuss any limitations of the review processes used.	Discussion on challenges and gaps
	23d	Discuss implications of the results for practice, policy, and future research.	Discussion on challenges and gaps
**OTHER INFORMATION**			
**Registration and protocol**	24a	Provide registration information for the review, including register name and registration number, or state that the review was not registered.	This review was not registered

Topic	No.	Item	Location where item is reported
	24b	Indicate where the review protocol can be accessed, or state that a protocol was not prepared.	No protocol was prepared for this review
	24c	Describe and explain any amendments to information provided at registration or in the protocol.	No protocol was prepared for this review
**Support**	25	Describe sources of financial or non-financial support for the review, and the role of the funders or sponsors in the review.	No protocol was prepared for this review
**Competing interests**	26	Declare any competing interests of review authors.	Acknowledgment
**Availability of data, code and other materials**	27	Report which of the following are publicly available and where they can be found: template data collection forms; data extracted from included studies; data used for all analyses; analytic code; any other materials used in the review.	See the Multimedia Appendix

From: Page MJ, McKenzie JE, Bossuyt PM, Boutron I, Hoffmann TC, Mulrow CD, et al. The PRISMA 2020 statement: an updated guideline for reporting systematic reviews. MetaArXiv. 2020, September 14. DOI: 10.31222/osf.io/v7gm2. For more information, visit: www.prisma-statement.org

PRIMSA Abstract Checklist

Topic	No.	Item	Reported?
**TITLE**			
**Title**	1	Identify the report as a systematic review.	Yes
**BACKGROUND**			
**Objectives**	2	Provide an explicit statement of the main objective(s) or question(s) the review addresses.	Yes
**METHODS**			
**Eligibility criteria**	3	Specify the inclusion and exclusion criteria for the review.	Yes
**Information sources**	4	Specify the information sources (e.g. databases, registers) used to identify studies and the date when each was last searched.	Yes

Topic	No.	Item	Reported?
**Risk of bias**	5	Specify the methods used to assess risk of bias in the included studies.	Yes
**Synthesis of results**	6	Specify the methods used to present and synthesize results.	Yes
**RESULTS**			
**Included studies**	7	Give the total number of included studies and participants and summarise relevant characteristics of studies.	Yes
**Synthesis of results**	8	Present results for main outcomes, preferably indicating the number of included studies and participants for each. If meta-analysis was done, report the summary estimate and confidence/credible interval. If comparing groups, indicate the direction of the effect (i.e. which group is favoured).	Yes
**DISCUSSION**			
**Limitations of evidence**	9	Provide a brief summary of the limitations of the evidence included in the review (e.g. study risk of bias, inconsistency and imprecision).	Yes
**Interpretation**	10	Provide a general interpretation of the results and important implications.	Yes
**OTHER**			
**Funding**	11	Specify the primary source of funding for the review.	No
**Registration**	12	Provide the register name and registration number.	No

From: Page MJ, McKenzie JE, Bossuyt PM, Boutron I, Hoffmann TC, Mulrow CD, et al. The PRISMA 2020 statement: an updated guideline for reporting systematic reviews. MetaArXiv. 2020, September 14. DOI: 10.31222/osf.io/v7gm2. For more information, visit: www.prisma-statement.org
